# Comparison of Fatty Acid Contents in Major Lipid Classes of Seven Salmonid Species from Siberian Arctic Lakes

**DOI:** 10.3390/biom10030419

**Published:** 2020-03-08

**Authors:** Nadezhda N. Sushchik, Olesia N. Makhutova, Anastasia E. Rudchenko, Larisa A. Glushchenko, Svetlana P. Shulepina, Anzhelika A. Kolmakova, Michail I. Gladyshev

**Affiliations:** 1 Institute of Biophysics of Federal Research Center “Krasnoyarsk Science Center” of Siberian Branch of Russian Academy of Sciences, Akademgorodok, 50/50, Krasnoyarsk 660036, Russia; makhutova@ibp.krasn.ru (O.N.M.); rudchenko.a.e@gmail.com (A.E.R.); angelika_@inbox.ru (A.A.K.); glad@ibp.ru (M.I.G.); 2 Siberian Federal University, Svobodny av., 79, Krasnoyarsk 660041, Russia; loraglushchenko@gmail.com (L.A.G.); shulepina@mail.ru (S.P.S.)

**Keywords:** arctic, Salmoniformes, long-chain polyunsaturated fatty acids, polar lipids, triacylglycerols, eicosapentaenoic acid, docosahexaenoic acid

## Abstract

Long-chain omega-3 polyunsaturated fatty acids (LC-PUFA) essential for human nutrition are mostly obtained from wild-caught fish. To sustain the LC-PUFA supply from natural populations, one needs to know how environmental and intrinsic factors affect fish fatty acid (FA) profiles and contents. We studied seven Salmoniformes species from two arctic lakes. We aimed to estimate differences in the FA composition of total lipids and two major lipid classes, polar lipids (PL) and triacylglycerols (TAG), among the species and to evaluate LC-PUFA contents corresponding to PL and TAG in muscles. Fatty acid profiles of PL and TAG in all species were characterized by the prevalence of omega-3 LC-PUFA and C16-C18 monoenoic FA, respectively. Fish with similar feeding spectra were identified similarly in multivariate analyses of total lipids, TAG and PL, due to differences in levels of mostly the same FA. Thus, the suitability of both TAG and total lipids for the identification of the feeding spectra of fish was confirmed. All species had similar content of LC-PUFA esterified as PL, 1.9–3.5 mg g^−1^, while the content of the TAG form strongly varied, from 0.9 to 9.8 mg g^−1^. The LC-PUFA-rich fish species accumulated these valuable compounds predominately in the TAG form.

## 1. Introduction

Long-chain omega-3 polyunsaturated fatty acids (LC-PUFA), such as eicosapentaenoic acid (EPA) and docosahexaenoic acid (DHA), are known to be essential compounds for human nutrition, since they can modulate the functioning of cardiovascular and neural systems and general metabolism, being the precursors for the synthesis of diverse lipid mediators and directly affecting membrane properties [1,2,3,4,5]. Most international and national health agencies and foundations recommended personal consumption of 0.5–1.0 g of EPA+DHA per day for reducing the risk of cardiovascular diseases and other metabolic disorders [6,7,8]. Although a lot of potential sources of LC-PUFA are now being considered, natural fish populations are still the major source of these compounds for human nutrition [9,10]. Recent reviews showed the deficiency of the LC-PUFA supply with fish caught from natural populations and emphasized the potential negative influence of some global threats, like climate change, pollution, eutrophication, etc. [10,11,12,13]. To challenge above threats and to sustain the LC-PUFA supply from natural populations, one needs to know how environmental and intrinsic factors affect fish fatty acid profiles and content, including those of EPA and DHA. Causes of variations of fatty acid composition and content in wild fish are still incompletely understood [10,14].

The ability of fish to deposit fat (lipids) in muscles varies from species to species and may be a crucial intrinsic factor [15]. According to their functions, lipids in fish, like in other animals, could roughly be divided into energy-reserve and membrane-structural groups [16,17]. Fish reserve lipids are primarily represented by triacylglycerols (TAG) and include mostly fatty acids that come from food sources. Fatty acid profiles of TAG are generally considered as valuable trophic markers due to their resemblance with fatty acid profiles of particular food sources [18]. In addition, the TAG fraction in fish can also contain high levels of monoenoic C16-C18 fatty acids that are intensively synthesized in so called “fatty” fish species to provide energy reserves. TAG molecules are accumulated either directly in muscle cells as droplets or in specific adipocytes which may be integrated in muscle tissues or form separate layers of adipose tissue.

The structural polar lipids (PL) that form fish cellular and intracellular membranes mostly comprise phospholipids [19,20]. As known, fatty acid composition of PL affects physico-chemical properties of cellular membranes. Hence, PL are considered to have conservative fatty acid profiles which slightly reflect that of diet. The essential omega-3 LC-PUFA are preferentially accumulated in PL fraction of muscle tissues due to their strong membrane-modulating properties. Thus, fatty acid profiles of the major lipid classes, TAG and PL, in fish muscles are different in general [19].

TAG content per mass unit of fish muscles is highly variable due to influence of many factors [15]. In contrast, PL content per mass unit of muscles is fairly constant [21]. Thereby, we hypothesize that PL specific content has a putative upper threshold, because amounts of PL molecules in tissue are likely determined by a volume of membranes.

Contents of omega-3 LC-PUFA in muscle tissue of diverse fish species greatly vary, approximately ~400-fold [22]. The question arises what part of this variation in total EPA and DHA contents is provided by TAG or PL variability? There is a basic assumption in the current literature that a major part of omega-3 LC-PUFA presents as acyl groups of membrane phospholipid molecules [11].

To evaluate contribution of the two major lipid fractions in total content of LC-PUFA in edible muscle tissue (filets) we studied seven commercial species of the order Salmoniformes that inhabit oligotrophic non-polluted lakes in Arctic Siberia. The fish species vary in their feeding habits and habitats and have different fat content in filets. Using data on these fish we aimed to compare distribution of fatty acids, including omega-3 LC-PUFA in total lipids and two major lipid classes: TAG and PL. Specifically, we aimed (i) to check if the fish species with various feeding spectra can be differentiated basing on FA profiles of total lipids, TAG or PL, (ii) to evaluate LC-PUFA content corresponded to TAG and PL classes in muscles, (iii) to range species according to their nutritive value for humans in respect of LC-PUFA content.

## 2. Materials and Methods

### 2.1. Sampling

Fish specimen of commercial sizes were collected during July 2017 from catches of local authorized fishers. Following sampling was done in accordance with the BioEthics Protocol on Animal Care approved by Siberian Federal University. The catches were from two oligotrophic arctic lakes, Sobachye and Pyasino. Sobachye Lake was previously characterized elsewhere [23]. Briefly, it is located at 69°01′ N 91°05′ E and has maximum depth of 162 m and area equal to 99 km^2^. Pyasino Lake situates at 69°40′ N 87°51′ E, has average depth of 4 m and an area equal to 735 km^2^ [24].

Whitefish *Coregonus lavaretus* (Linnaeus, 1758), non-identified form of whitefish *C. lavaretus*, round whitefish *Prosopium cylindraceum* (Pennant, 1784) and charr *Salvelinus drjagini* Logashev, 1940 were caught in Sobachye Lake; broad whitefish *Coregonus nasus* (Pallas, 1776), muksun *Coregonus muksun* (Pallas, 1814) and inconnu *Stenodus leucichthys nelma* (Guldenstadt, 1772) were caught in Pyasino Lake. All studied fish species are of the Salmoniformes order. Numbers of samples, average individual sizes and weights, and main food sources for the studied fish species are given in Table 1. Stomach contents of all specimen were studied under a light microscope, and main food items were identified to a possible taxon level.

For biochemical analyses, we cut slices of fish white muscles of approximately 2–3 g, 2–3 cm below the dorsal fin. The samples were subdivided into two parts: for FA and moisture analyses. For FA analyses, ca. 1 g of muscle tissues was immediately placed into a volume of 3 mL of chloroform/methanol (2:1, by vol.) and kept until further analysis at −20 °C. Another subsample of ca. 1–2 g of wet weight was weighed, dried at 105 °C until constant weight, and weighed dry for moisture calculation.

### 2.2. Lipid and Fatty Acid Analyses

In laboratory, lipids were extracted with chloroform/methanol (2:1, by vol.) in triplicate, simultaneously with homogenizing tissues with glass beads in a mortar. Prior to the extraction, an aliquot of 19:0-fatty acid methyl ester (FAME) chloroform solution, as the internal standard, was added to samples for quantification of chromatographic peaks. The extracts were combined and dried with anhydrous Na_2_SO_4_ and the solvents were roto-evaporated under vacuum at 35 °C. The extracted lipids were redissolved in a 1 mL portion of chloroform and separated in two equal parts. To analyze the fatty acid composition of total lipids, one part of the lipid extract was methylated in the following way. The lipids were hydrolysed under reflux at 90° C for 10 min in 0.8 mL of methanolic sodium hydroxide solution (8 g/L). Then the mixture was cooled for 5 min at room temperature. Next, 1 mL of methanol/sulphuric acid (97:3, by vol.) was added and the mixture was heated under reflux at 90 °C for 10 min to methylate free fatty acids. At the end, 5 mL of saturated solution of NaCl and 3 mL of hexane were added. The FAMEs were extracted for 1 min, the mixture was transferred to a separatory funnel, and the lower aquatic layer was discarded. The hexane layer was additionally washed once with an aliquot of the NaCl solution and twice with 5 mL of distilled water. Then the hexane solution of FAMEs was dried with anhydrous Na_2_SO_4_, and hexane was removed by roto-evaporating at 30 °C.

We fractionated another part of the lipid extracts by thin layer chromatography (TLC) on silica gel G with hexane-diethyl ether-acetic acid mixture (85:15:1, by vol.). We prepared a reference mixture containing triolein, oleic acid, cholesterol, and phosphatidylcholine (Sigma, St. Louis, MO, USA), which was applied on the side lanes of the silica gel plates. To identify the lipid composition of fish species, we applied aliquots of samples to the plates, and then developed them as described above. After developing, the plates were sprayed with mixture of ethanol/sulphuric acid (90:10, by vol.) and gently heated until grey spots of lipid classes appeared. To evaluate fatty acid profiles and quantify dominant lipid classes, we separated a main portion of lipid extracts on the silica gel plates, and then visualized only the side lanes corresponding to the reference–compound mixture by a reaction with phosphomolybdic acid ethanolic solution. Lipid spots of the fish samples on the plates were blind detected according to positions of the reference compounds. The lipid spots containing TAG and PL fractions were scraped off from the silica gel plates and placed in flasks. Aliquots of 19:0-FAME hexane solution (t internal standard) were added into the flasks containing silica gel powder with the lipid fractions. To prepare FAMEs, 1ml of hexane and 0.2 mL of fresh 3 M sodium methoxide methanolic solution was added, and the mixture was shaken vigorously for 1 min. Subsequently, the mixture was kept calm at room temperature for 5 min, and finally 3 mL of hexane and 5 mL of a saturated solution of NaCl were added. The next procedure of FAME extraction and washing was the same as for those prepared from total lipids.

Analyses of all FAMEs were done with a gas chromatograph equipped with a mass spectrometer detector (model 6890/5975C; Agilent Technologies, Santa Clara, CA, USA) and with a 30-m long, 0.25-mm internal diameter capillary HP-FFAP column. Detailed descriptions of the chromatographic and mass-spectrometric conditions are given in [23].

### 2.3. Statistical Analysis

The Kolmogorov–Smirnov one-sample test for normality *D*_K-S_, standard errors (SE), Student’s *t*-tests, one-way ANOVA with post hoc Tukey HSD test, Kruskal–Wallis test (in the absence of normal distribution) and canonical correspondence analysis (CCA) were calculated conventionally, using STATISTICA software, version 9.0 (StatSoft Inc., Tulsa, OK, USA).

## 3. Results

According to gut content analysis, *C. lavaretus* in Sobachye Lake was benthivorous (Table 1). Whitefish of a non-identified form in this lake consumed mostly pupa and adult insects, i.e., foraged near the water surface. Round whitefish fed on benthic invertebrates and algae (Table 1). Both broad whitefish and muksun in Pyasino Lake consumed benthic food items, including detritus. Charr in Sobachye Lake and inconnu in Pyasino Lake were piscivorous (Table 1).

Average values of moisture content, lipid content and sum of fatty acid content for total lipids in the studied fish are given in Table 2. Lower moisture values were characteristic of the species with higher values of lipid and sum FA content, charr and whitefish (Table 2). In contrast, round whitefish and whitefish of the non-identified form had the maximum moisture content and the minimal contents of lipids and sum FA. Sum FA content of total lipids significantly varied ~7-fold among the studied species (Table 2). Based on the averages of lipid and sum FA contents, charr and whitefish are further considered as “fatty” fish, muksun, inconnu and broad whitefish as “medium fat” fish, and round whitefish and the non-identified form of whitefish as “lean” fish (Table 2).

Levels of 25 prominent individual FA and their structural groups in total lipids are showed in Table 3. Charr had the highest levels of 20:2n-6, 20:3n-3, 20:4n-3, 22:4n-3 and C24 PUFA among the studied species (Table 3). Whitefish had the significantly highest levels of 16:1n-7 and C16 PUFA, and tended to be higher in levels of 18:1n-9 and 20:5n-3. Muksun tended to have higher levels of 14:0, 20:1 and 22:5n-6 (Table 3). Broad whitefish had higher levels of 16:1n-9, C15-17 BFA (branched-chain fatty acids), 18:0, 18:1n-7, 18:2n-6, 18:3n-3 (Table 3). Whitefish of the non-identified form had higher levels of 16:0 and 22:6n-3 compared to those of the other fish. Inconnu and round whitefish had intermediate levels of all FA in total lipids (Table 3).

**Figure 1:**
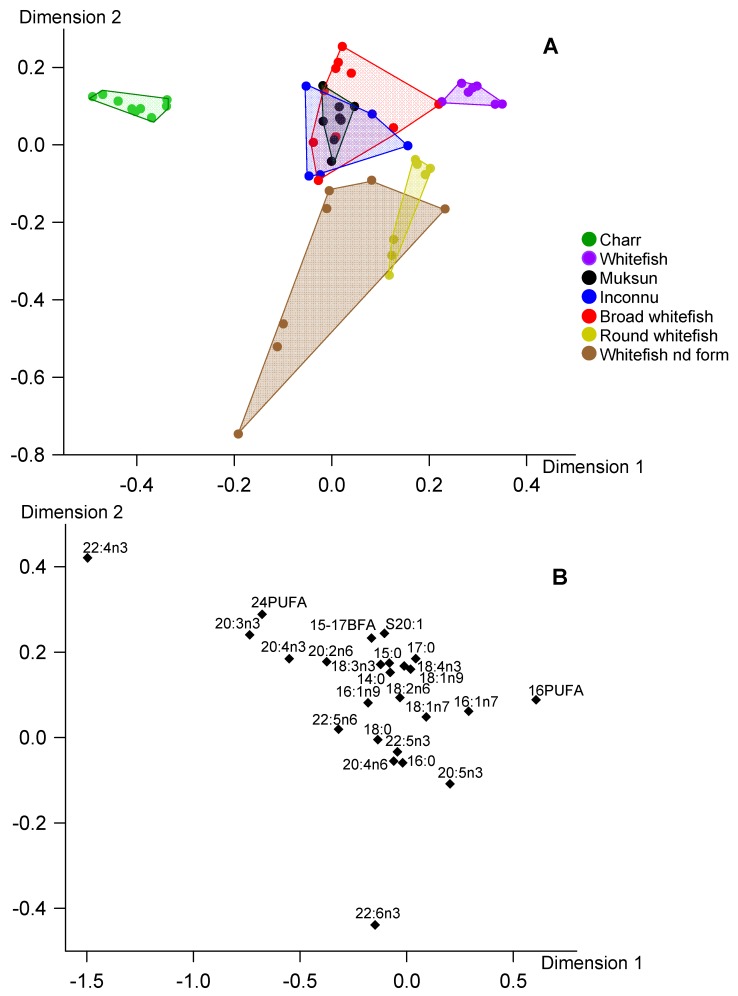
Canonical correspondence analysis of fatty acid percentages (% of FA sum) in total lipids of muscles of seven fish species from arctic lakes (Siberia, Russia). **A**—individual variables, **B**—factor structure coefficients for fatty acids. Dimension 1 and Dimension 2 represented 33.9% and 21.2% of inertia, respectively.

We performed CCA of FA profiles of total lipids of the fish species to find out their overall differences (Figure 1). Along Dimension 1, most difference in FA composition was observed between charr, on the one hand, and whitefish, on the other hand. The differences were primarily caused by higher percentages of 22:4n-3, 20:3n-3 and C24 PUFA in charr, and higher percentages of C16 PUFA and 16:1n-7 in whitefish. In Dimension 2, whitefish of a non-identified form and round whitefish located at the one end and broad whitefish was at the other end (Figure 1). Whitefish of the non-identified form and round whitefish were separated due to higher levels of 22:6n-3 and 20:5n-3, and partial separation of broad whitefish was due to higher levels of C15-17 BFA (Table 3). Samples of the non-identified whitefish were markedly scattered (Figure 1) due to the high variability in percentages of 22:6n-3 (Table 3).

In PL of all studied species, 22:6n-3, 16:0 and 20:5n-3 were dominant fatty acids (Table 4). Charr had the highest levels of 22:6n-3, 22:4n-3, 22:5n-6, 20:4n-3 among the studied species (Table 4). Whitefish had a significantly higher level of 20:5n-3 compared to that of the other fish. Inconnu tended to be higher in 18:1n-9 level. Broad whitefish had the highest levels of 20:4n-6 and 18:2n-6 (Table 4). Round whitefish had significantly higher levels of 16:1n-7, 18:1n-7 and C16 PUFA compared to those of the other fish. Muksun and whitefish of the non-identified form had intermediate levels of most FA in PL (Table 4).

**Figure 2:**
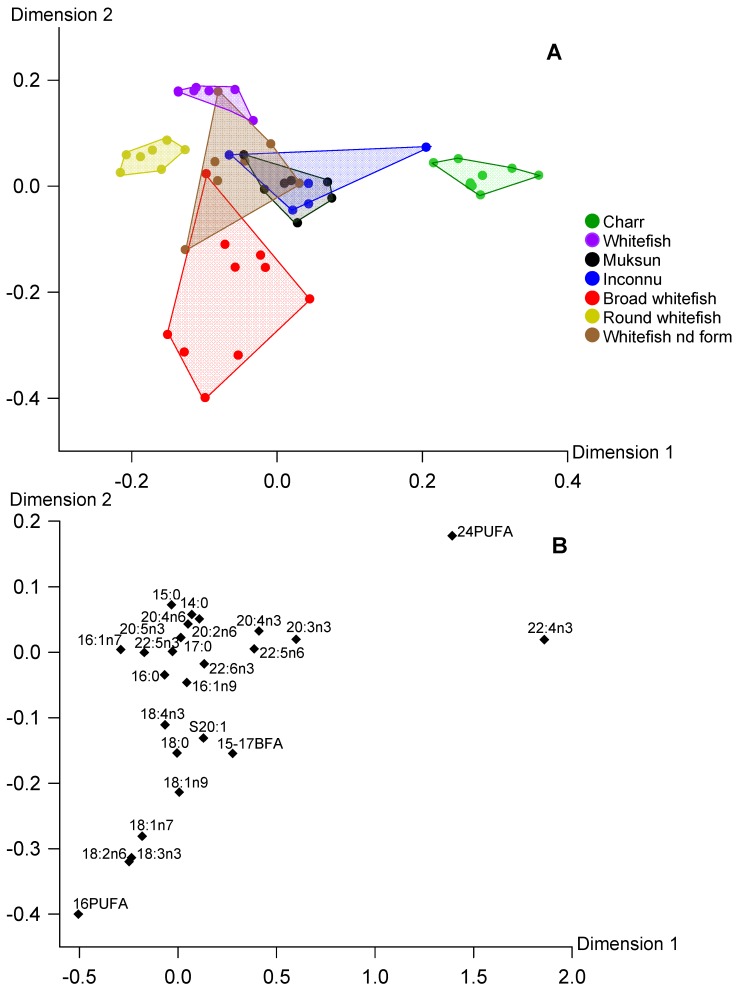
Canonical correspondence analysis of fatty acid percentages (% of FA sum) in polar lipids of muscles of seven fish species from arctic lakes (Siberia, Russia). **A**—individual variables, **B**—factor structure coefficients for fatty acids. Dimension 1 and Dimension 2 represented 30.9% and 22.5% of inertia, respectively.

To reveal overall differences in PL FA, CCA was performed (Figure 2). In the first dimension, a conspicuous difference of round whitefish versus charr was found. This difference was provided mostly by the greater levels of C16 PUFA, 16:1n-7 and 18:2n-6 in PL of round whitefish and by greater levels of C24 PUFA and 22:4n-3 in that of charr (Figure 2). The variation in the second dimension of CCA was related to differences between whitefish and broad whitefish due to levels of 15:0 and 14:0 versus levels of C16 PUFA, 18:2n-6 and 18:3n-3.

In FA composition of triacylglycerols of all the studied arctic fish, 18:1n-9, 16:1n-7, 16:0 and 20:5n-3 dominated (Table 5). Charr had the highest levels of 22:6n-3, C24 PUFA, 22:5n-3, 20:4n-3 and 20:3n-3 compared to that of the other studied species. Whitefish had the maximum levels of 18:1n-9 and 20:5n-3 (Table 5). Muksun had the significantly higher level of 14:0 than the other fish. Inconnu and whitefish of the non-identified form had intermediate FA levels in TAG (Table 5). Broad whitefish had the maximum levels of 16:0, C15-17 BFA, 17:0, 18:2n-6, 18:3n-3, and 20:4n-6 among the studied fish. Round whitefish had the significantly higher percentages of 16:1n-7 and C16 PUFA in TAG (Table 5).

**Figure 3:**
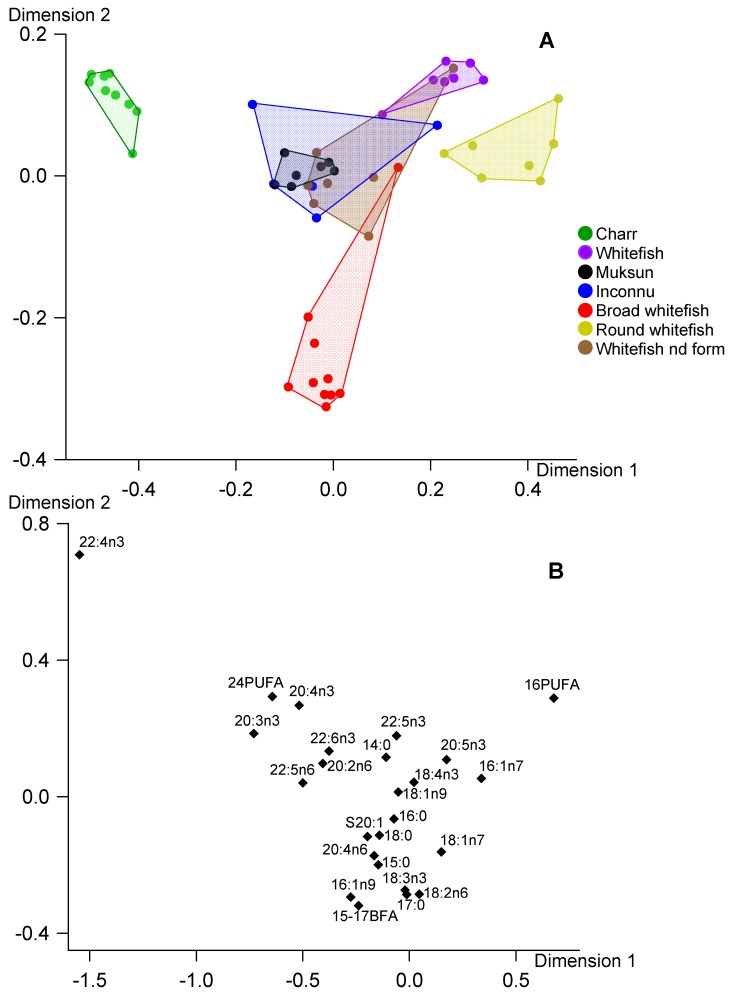
Canonical correspondence analysis of fatty acid percentages (% of FA sum) in triacylglycerols of muscles of seven fish species from arctic lakes (Siberia, Russia). **A**—individual variables, **B**—factor structure coefficients for fatty acids. Dimension 1 and Dimension 2 represented 51.1% and 15.6% of inertia, respectively.

To study differences in fish reserve lipids, we performed CCA of FA in TAG (Figure 3). Like the multidimensional analysis for PL, Dimension 1 showed a marked difference of round whitefish versus charr. This difference was provided mostly by the greater levels of C16 PUFA and 16:1n-7 in TAG of round whitefish and by greater levels of 22:4n-3 and 20:3n-3 in that of charr (Figure 3). The second dimension of CCA for TAG also showed a similar trend to that observed in CCA of PL (Figure 2 and Figure 3). In this dimension, most prominent difference was found between whitefish and broad whitefish due to variability in levels of C15-17 BFA, 17:0 and C16 PUFA (Figure 3).

In general, positioning of fish species in the biplot for reserve TAG well corresponded to that in biplot for structural PL (Figure 2 and Figure 3). It should be also noted that physiologically significant EPA and DHA were not found among the FA markers responsible for separation of fish species in CCA for TAG and PL (Figure 2 and Figure 3). Positioning of fish species in the biplot for total lipids generally corresponded to that in biplots for the lipid classes, with exception of whitefish of the non-identified form (Figure 1, Figure 2 and Figure 3). The fatty acid markers responsible for separation of the fish samples in CCA were generally similar for total lipids, TAG and PL with exception of DHA and EPA in CCA of total lipids (Figure 1, Figure 2 and Figure 3).

**Figure 4:**
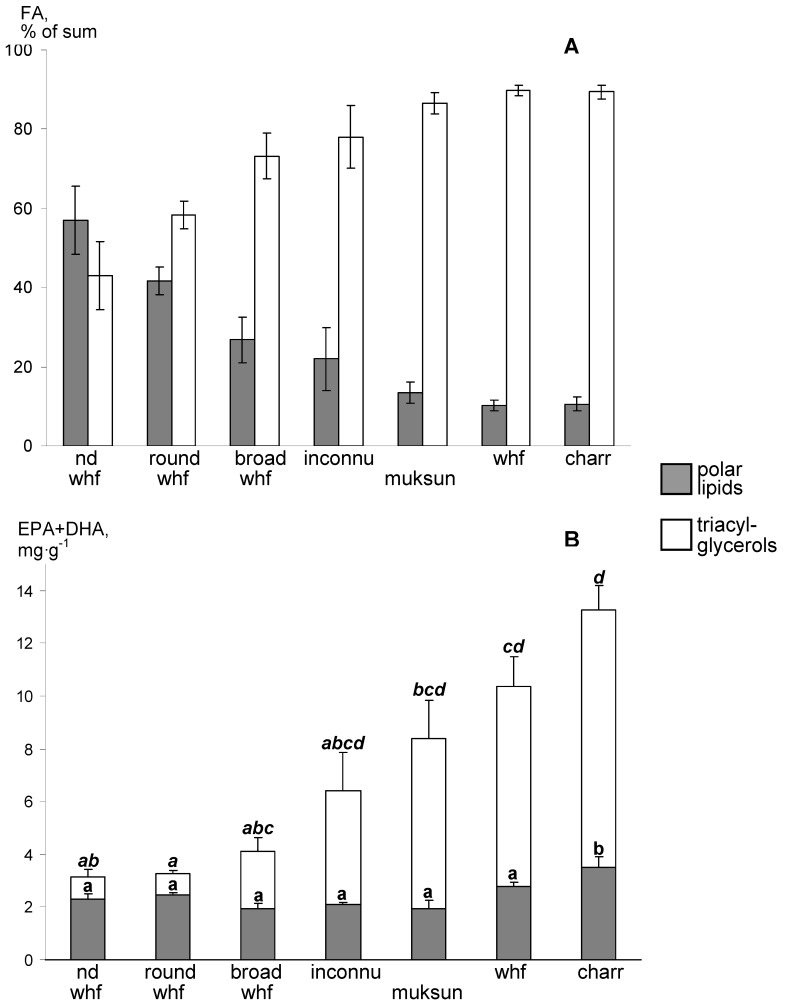
Mean percentages of polar lipids and triacylglycerols of their sum—**A**, and mean contents (mg g^−1^ wet weight) of sum of eicosapentaenoic and docosahexaenoic acids that corresponded to polar lipids and triacylglycerols—**B**, in muscles of seven fish species from arctic lakes (Siberia, Russia): nd whf—the non-identified form of *C. lavaretus* from Sobachye Lake, round whf—*P. cylindraceum* from Sobachye Lake, broad whf—*C. nasus* from Pyasino Lake, inconnu—*S. leucichthys nelma* from Pyasino Lake, muksun—*C. muksun* from Pyasino Lake, whf—*C. lavaretus* from Sobachye Lake, charr—*S. drjagini* from Sobachye Lake. Bars represent standard errors. Means labelled with the same letter are not significantly different at *P* < 0.05 after Tukey HSD post hoc test.

A visual analysis of all thin-layer chromatograms showed a marked dominance of TAG, PL and sterols as major lipid fractions. Spots that corresponded to other lipid classes were negligible. Therefore, we considered TAG and PL as major acyl-containing fractions, summarized their FA contents per mass unit (Table 4 and Table 5) and calculated their parts in the sum of FA in the fish muscles (Figure 4A). Polar lipids constituted from 10.3 to 57.0% of the acyl-containing lipid sum, being the highest in whitefish of the non-identified form (Figure 4A). Triacylglycerols were the dominant acyl-containing lipid fraction for majority of the studied fish and exceeded 85% in charr, whitefish and muksun (Figure 4A). Note that increase in lipid content and total fatty acids for the studied species well corresponded with the increase in the TAG proportion of the acyl-containing lipids (Table 2, Figure 4A).

Using the PL and TAG percentages of the acyl-containing lipid sum and content of EPA and DHA of total lipids per mass unit, we calculated parts of EPA + DHA that provided by polar lipids versus triacylglycerols and expressed them as mg g^−1^ wet weight of muscle tissue (Figure 4B). Contents of EPA + DHA provided by PL fraction varied from 1.9 to 3.5 mg g^−1^ (Figure 4B). The average value for the seven fish species was 2.4 ± 0.2 mg g^−1^, and coefficient of variation, CV, was 8.7%. Contents of EPA + DHA in TAG were of a greater range, from 0.9 to 9.8 mg g^−1^; the average value was to 4.4 ± 0.2 mg g^−1^, and CV was 28.9% (Figure 4B). 

## 4. Discussion

### 4.1. Main Finding

All the taxonomically related species of order Salmoniformes had nearly similar content of EPA+DHA in PL, 2.4 ± 0.2 mg g^−1^, in average. In contrast, content of EPA + DHA esterified as TAG varied ~10-fold among the studied salmonids. Thus, all variations of nutritive value, i.e., EPA + DHA content per mass unit of filet, were caused by TAG fraction, while PL had a constant species (taxon—specific physiologically optimum content. As a result, fatty fish, charr, whitefish and muksun, contained most amounts of nutritionally valuable EPA and DHA in TAG fraction of muscles. Conversely, the lean species, round whitefish and whitefish of the non-identified form, had the omega-3 LC-PUFA contained mostly in polar lipids. Regarding nutritive value, the fatty species with higher proportion of TAG in muscles, charr, whitefish and muksun, appeared to be most valuable and had 13.3 ± 0.8, 10.4 ± 1.1 and 8.4 ± 1.4 mg g^−1^, respectively.

### 4.2. Fatty Acid Markers in Fish Total Lipids, TAG and PL

In our snap-shot field study of salmonids from arctic lakes we found significant differences between fatty acid profiles of the main lipid classes, with prevalence of n-3 LC-PUFA and monoenoic 16-18 FA in structural PL and storage TAG, respectively. In addition, both FA profiles of TAG and PL, as well as profiles of total lipids, had distinct peculiarities among the studied fish that allowed separating the most species in the CCA biplots (Figure 1, Figure 2 and Figure 3). It should be emphasized that the separations of the fish in the multivariate analyses of all three lipid fractions were provided mainly by the same marker FA: C24 PUFA, 22:4n-3, 20:3n-3, C16 PUFA, 16:1n-7 and C15-17 BFA. The conspicuous exception was 22:6n-3 and 20:5n-3 in total lipid CCA that separated whitefish of the non-identified form and round whitefish from the other species (Figure 1). PL had a predominant contribution to total lipids of these lean fish (Figure 4A). Therefore, FA profiles of total lipids of whitefish of the non-identified form and round whitefish mostly reflected FA composition of PL which were considerably rich in DHA and EPA (Table 4). As a result, DHA and EPA, dominant FA of PL, played as markers for the lean fish in CCA of total lipids.

Similar patterns of biomarker FA, characteristic of zoobenthic, algal, terrestrial and other food sources, within TAG and PL fractions of the studied species allowed to use both these fractions, as well as their sum, total lipids, for identification of food sources of wild fish (Figure 1, Figure 2 and Figure 3). In ecological studies, analysis of FA trophic markers of various consumers is often performed for TAG assuming that they generally deposit fatty acid molecules coming from food assimilation [18,25,26]. Meanwhile, many studies used FA composition of total lipids to elucidate trophic relations of various fish species, e.g., [27,28,29,30,31]. In overall, both approaches base on a premise that biochemical composition of food sources is reflected by FA profiles of TAG or total lipids.

Another lipid class of high concern is polar lipids comprised mostly phospholipids and glycolipids that are main constituents of cell membranes. As known, the specific FA composition of PL provides proper membrane structure and functions. As a result, FA composition of PL are considered to be highly conserved relative to diet and tended to reflect FA biosynthetic capacities of an organism [18].

In contrast to the common opinion on conserved PL composition, in some studies FA profiles of PL were successfully used as trophic markers. For instance, fatty acid profiles of both polar and neutral lipids conspicuously differed among three species, benthivorous whitefish *Coregonus clupeaformis*, and piscivorous walleye *Sander vitreus* and northern pike *Esox lucius*, due to different feeding habits of the fish [32]. We also confirmed that PL FA profiles of muscles allow to identify feeding spectra of fish similarly that TAG profiles do (Figure 2 and Figure 3). For instance, round whitefish was one of the most separated species in the both multivariate analyses of TAG and PL due to the greatest levels of C16 PUFA and 16:1n-7 which originated from diatoms and green algae [33]. Indeed, the algae were one of the dominant items in stomach content of this species (Table 1).

In CCA biplots of total lipids, PL and TAG, charr had a particular position due to higher levels of minor n-3 PUFA, like 22:4-3, C24 PUFA and 20:3n-3. These FA were not assigned as trophic markers, whereas some of them, C24 PUFA, were considered as intermediate compounds indicative for conversion of C20 to C22 PUFA [34,35]. In TAG of charr the percentage of 22:4n-3 accounted for 1.3% of FA sum, being absent or found in traces in other studied fish. The presence of this PUFA was previously reported for least cisco *Coregonus sardinella*, small-sized pelagic fish inhabited Sobachye Lake [23]. The studied charr from Sobachye Lake was piscivorous (Table 1), thus, it could obtain this PUFA from the consumed least cisco. Like in our study, species of the same genus and its prey, lake trout *Salvelinus namaycush* and cisco from Great Bear Lake, were together separated from other hydrobionts in a multivariate analysis due to higher levels of 22:4-3 and 20:3n-3 [31]. Alternative explanation based on coincidence between 22:4n-3 and C24 PUFA levels is that the fatty acid 22:4n-3 may be a marker of LC-PUFA conversion in fish. Anyway, we suppose that considerable levels of 22:4n-3, 20:3n-3 and C24 PUFA might be a characteristic feature of FA profiles of *Salvelinus* genus.

In both CCA analyses of TAG and PL, broad whitefish well separated from the other species due to higher levels of C15-17 BFA, 17:0, 18:2n-6 and 18:3n-3. The two former FA are known to be markers of bacterial organic matter, while the two latter are considered as markers of terrestrial organic matter [31,33]. Broad whitefish is a typical benthivorous species that likely got these marker fatty acids from detritus enriched with bacterial and terrestrial organic matter. The species had the highest levels of 18:2n-6 in TAG, and 20:4n-6 in PL, relatively. This finding likely indicates for the initial storage of dietary 18:2n-6 in TAG and its consequent conversion to 20:4n-6 with further transfer to PL.

The similarity of FA sets that are markers for food sources between TAG and PL classes likely indicates that the studied wild fish are able to directly incorporate dietary biochemical components, i.e., fatty acyl groups, into membrane PL. Besides, FA originated from food assimilation, fish are able to include in lipid molecules fatty acyl groups obtained due to biosynthesis or conversion from precursors. Freshwater fish are known to have capacity to synthesize LC-PUFA from the shorter chain precursors [35,36]. Indeed, some studied fish, e.g., charr, contained in TAG and PL certain amounts of C24 PUFA and 20:4n-3 that likely were intermediates of DHA and EPA synthesis.

### 4.3. Content of Essential LC-PUFA in Fish PL and TAG

The studied seven salmonid species varied ~ 7-fold in total lipid and fatty acid contents per a mass unit of muscle tissues. Most of this variation was related with different TAG content in muscles (Table 5, Figure 4), whereas PL content evaluated as their FA sum varied slightly (Table 4). The observed variation in lipid class contents in the studied fish is in agreement with well-known notion that polar lipids comprise cellular membranes and, as a result, have a relatively constant content in muscle cells, in contrast to that of triacylglycerols [19,21,37]. For instance, an absence of relation between total lipid and phospholipid contents and a strong relation between total lipid and triacylglycerol contents expressed as percentages of muscle mass were previously shown for a number of marine myctophid species [38].

Fish polar lipids are commonly considered as a physiologically crucial lipid class that are rich in LC-PUFA, mostly in DHA and, to a lesser extent, in EPA [19,37,39]. Indeed, percentages of DHA and EPA in PL of various wild marine and freshwater species ranged as 11.5–55.7% and 2.6–14.6%, with average values of 31.4% and 7.4%, respectively [40,41,42,43,44,45,46]. The average levels of EPA and DHA in the fish species from our study coincided with the above ranges, except the EPA value of whitefish, 16.6%, which was a bit higher than the known values.

Triacylglycerols are considered to be relatively poor in LC-PUFA and preferably accumulate monoenoic C16-22 FA [18,19,37]. According to the available data, levels of DHA and EPA in fish TAG varied in intervals of 2.3%-23.3% and 1.1%–14.1%, with average values of 8.8% and 5.3%, respectively [40,41,42,43,44,45,46]. The percentages of both EPA and DHA of TAG in the fish species from our study well coincided with the above ranges (Table 5).

Triacylglycerols commonly comprise a large part of total lipids in muscles of medium-fat and fatty fish species. For instance, TAG achieved 80%, 90% and 51.5% in marine species arrow-tooth flounder (*Atheresthes stomias)* and golden pompano (*Trachinotus blochii*) and freshwater whitefish (*Coregonus lavaretus*), respectively [43,47,48]. In the studied freshwater salmonids, TAG percentages varied from 43.4% to 89.7% of the sum of two acyl-containing lipid classes (Figure 4A). Such high TAG levels may be explained by adaptation of the fish species to low-temperature conditions in the studied arctic lakes [49,50].

Regarding the relatively high contents of TAG per mass unit and percentages of EPA and DHA in TAG, we hypothesized that content of EPA and DHA in TAG would appreciably contribute to total content of EPA and DHA and would increase along with lipid content in muscles of the studied fish. Hence, we compared content of EPA and DHA esterified as TAG versus that esterified as PL. Content of EPA+DHA in PL per mass unit of muscle tissues were similar among the studied salmonids, moderately varying in the interval of 1.9–3.5 mg g^−1^ (Figure 4B). In contrast, values of EPA+DHA in TAG of the fish species greatly varied, ~10-fold. Lean fish, i.e., whitefish of the non-identified form, round whitefish and broad whitefish, contained only 25%–47% of EPA + DHA of total content of these PUFA in the muscles esterified as TAG molecules. In contrast, the medium-fat and fatty fish, inconnu, muksun, whitefish and charr, had more than half of their muscle EPA and DHA content as TAG molecules, up to 72%. Thus, the wild salmonids that had relatively high content of n-3 LC-PUFA in muscles (~ > 5 mg g^−1^) contained the major portion of these nutritionally valuable compounds in the storage lipids. Our finding evidently contradicts a common notion that lean and medium-fat fish that have PL as a main lipid class in the muscles are the best dietary sources of n-3 LC-PUFA for humans [19,51]. Wild fatty fish which are able to deposit large amounts of storage lipids in their muscles appear to be the most valuable sources of n-3 LC-PUFA in human diet.

Further, our results are in a good accordance with many studies showed strong relationship between total lipid and EPA, DHA or their sum contents in fish muscles. Such relation was found for marine species, sprat *Sprattus sprattus* and herring *Clupea harengus* from Baltic Sea [52], for five marine species from the northeast Pacific [38] and for several freshwater species from a subalpine lake [53]. The significant relation was also found across farmed families of Atlantic salmon *Salmo salar* [54].

It is interesting to note that percentages of the n-3 LC-PUFA and lipid (or total FA as its proxy) content were negatively correlated in aforementioned and other studies [10]. The reported negative correlation was explained by the fact that total lipids increase preferably at the expense of TAG, whereas content of the membrane phospholipids, which are rich in n-3 PUFA remains fairly constant [21,37]. As a result, the proportions of EPA and DHA in muscle total lipids become diluted due to the accumulation of neutral lipids, which have high levels of monounsaturated FA. Although increase of total lipid content at the expense of TAG in fish muscles, as a rule, leads to decrease of n-3 LC-PUFA percentage, this does not mean that a concomitant decrease of nutritional quality of a fish occurs. Nutritional quality of fish products must be estimated on quantitative base expressed as mg FA per gram of tissue rather than percentage base [10].

Quantitative (mg per gram of tissue) measurements of TAG versus PL contribution in n-3 LC-PUFA of fish muscles are very scarce. Some studies gave indirect evidence of significant TAG contribution. For instance, among four fish species commercially harvested in Alaskan waters, arrow-tooth flounder *A. stomias* had maximum contents of EPA and DHA, 7.0 mg g^−1^, as well as maximum levels of TAG, 80% of total lipids in edible muscles [47]. Myctophid fish species with higher total lipid content (proxy for TAG content) also had higher contents of EPA and DHA esterified as TAG [38].

Some direct measurements showed that lean fish contained less than half of EPA and DHA in their muscles esterified as TAG, e.g., wild white seabream *Diplodus sargus* [40], whitefish *Coregonus lavaretus* [43], six commercial Chilean marine species [46]. In contrast, the only studied medium-fat fish (2–4% lipid content of wet mass), Pacific sandperch *Prolatilus jugularis*, had approximately 60% of EPA+DHA esterified as TAG [46]. Similar to latter finding, farmed *C. lavaretus* which had one of the highest known values of EPA and DHA in muscles, 18.6 mg g^−1^ wet weight, had 61% of that value in TAG [43]. In our study, the fish species were strongly variable in lipid and total FA content and, as a result, in n-3 LC-PUFA content esterified as TAG. Like in the abovementioned studies, the fatty fish, muksun, whitefish and charr, had relatively higher content of EPA + DHA per mass unit that were mostly esterified as TAG (Figure 4B).

If we take the threshold of the recommended personal daily dose of EPA + DHA as 1 g and the average per serve portion of fish as 200 g [55,56], a fish of proper nutritional value should contain EPA + DHA nearly or more than 5 mg g^−1^ of filet [57]. The obtained data on lipid class composition and content mean that when such fish is consumed, nearly or more than half of the essential n-3 LC-PUFA comes as TAG form. Recent studies showed that bioavailability of FA, including LC-PUFA esterified as TAG may be lower than that esterified as PL [58,59], but see [60]. Thus, distribution of LC-PUFA in major lipid classes should be further addressed in studies of nutritional quality of various fish products.

## 5. Conclusions

The studied fish with similar feeding spectra were identified similarly by a multivariate analysis of FA profiles of total lipids, TAG and PL. Marker FA characteristic of diverse food sources (benthic, terrestrial, etc.), accumulated in nearly similar proportions within TAG and PL, and thereby allow to use both these fractions, as well as total lipids, for identification of food sources of wild fish. The found incorporation of the fatty acid trophic markers in structural polar lipids similarly to that in reserve TAG deserves further studies. Regarding contribution of TAG and PL into content of essential LC-PUFA of the taxonomically closely related fish species of order Salmoniformes, we found that content of EPA+DHA esterified as PL was nearly invariable, presenting presumably a species/taxon-specific optimal level. In contrast, content of EPA+DHA esterified as TAG greatly varied among the studied fish and provided most contribution to total EPA+DHA content in the fatty fish species, charr, whitefish and muksun. We can conclude that EPA+DHA-rich fish species likely accumulate these nutritionally valuable compounds predominately in the TAG form.

## Figures and Tables

**Table 1:** The basic biological and sampling information on fish species (Salmoniformes order) from Siberian arctic lakes, 2017: *n*—number of sampled individuals; L—total length, cm (mean ± SE); W—total weight, g (mean ± SE); Food—items found in stomachs.

Common and Species Name	Lake	*n*	L	W	Food
Charr *Salvelinus drjagini*	Sobachye	9	608 ± 17	2371 ± 271	Fish (salmonids)
Whitefish *Coregonus lavaretus*	Sobachye	7	480 ± 23	1153 ± 167	Amphipods, mollusks, chironomid larvae
Muksun *Coregonus muksun*	Pyasino	8	492 ± 14	1271 ± 160	Ostracods, mollusks, chironomid larvae, detritus
Inconnu *Stenodus leucichthys nelma*	Pyasino	5	675 ± 86	3239 ± 1581	Fish
Broad whitefish *Coregonus nasus*	Pyasino	10	563 ± 17	1916 ± 183	Gastropods, detritus
Round whitefish *Prosopium cylindraceum*	Sobachye	7	409 ± 7	488 ± 27	Caddisfly and chironomid larvae, filamentous algae
Whitefish *Coregonus lavaretus* non-identified form	Sobachye	7	402 ± 12	568 ± 80	Chironomid and other insect pupa and adults

**Table 2:** Average (± SE—standard errors) moisture content (% wet weight), lipid content (mg g^−1^ wet weight) and sum fatty acid content for total lipids (mg g^−1^ wet weight) in muscle tissues of fish species caught in Siberian arctic lakes, 2017. Means of total fatty acids labeled with the same letter are not significantly different at *P* < 0.05 after ANOVA post hoc Tukey HSD test.

Common and Species Name	Moisture	Lipids	Total Fatty Acids
Charr *Salvelinus drjagini*	69.8 ± 1.3	155.8 ± 7.4	78.8 ± 5.1 ^D^
Whitefish *Coregonus lavaretus*	69.8 ± 1.4	82.0 ± 1.9	62.7 ± 7.2 ^CD^
Muksun *Coregonus muksun*	74.4 ± 1.0	n.d.	45.4 ± 8.7 ^BC^
Inconnu *Stenodus leucichthys nelma*	72.1 ± 2.6	68.4 ± 11.4	36.5 ± 10.0 ^ABC^
Broad whitefish *Coregonus nasus*	73.8 ± 1.4	n.d.	31.9 ± 5.1 ^AB^
Round whitefish *Prosopium cylindraceum*	76.1 ± 0.6	39.1 ± 3.3	13.8 ± 1.3 ^A^
Whitefish *Coregonus lavaretus* non-identified form	76.0 ± 0.9	41.1 ± 2.1	11.5 ± 1.9 ^A^

n.d.—no data.

**Table 3:** Mean levels of fatty acids (% of the total) in total lipids of species of Salmoniformes order: charr—*S. drjagini* from Sobachye Lake; whitefish—*C. lavaretus* from Sobachye Lake; muksun—*C. muksun* from Pyasino Lake; inconnu—*S. leucichthys nelma* from Pyasino Lake; broad whitefish—*C. nasus* from Pyasino Lake; round whitefish—*P. cylindraceum* from Sobachye Lake; whitefish nd—the non-identified form of *C. lavaretus* from Sobachye Lake. Cases (fatty acids) with normal distribution are given in bold. Means labeled with the same letter are not significantly different at *P* < 0.05 after ANOVA post hoc Tukey HSD test (cases with normal distribution) or Kruskal–Wallis test. If ANOVA is insignificant, letters are absent.

Fatty Acid	Charr	Whitefish	Muksun	Inconnu	Broad Whitefish	Round Whitefish	Whitefish nd
**14:0**	4.0 ± 0.1 ^A^	2.9 ± 0.1 ^B^	4.2 ± 0.2 ^A^	3.4 ± 0.1 ^AC^	2.5 ± 0.2 ^B^	2.2 ± 0.2 ^B^	2.3 ± 0.2 ^B^
**15:0**	0.3 ± 0.0 ^A^	0.2 ± 0.0 ^CD^	0.5 ± 0.0 ^B^	0.4 ± 0.0 ^A^	0.5 ± 0.0 ^B^	0.2 ± 0.0 ^D^	0.3 ± 0.0 ^C^
**16:0**	15.4 ± 0.2 ^C^	14.9 ± 0.2 ^C^	15.2 ± 0.2 ^C^	16.3 ± 0.7 ^AC^	18.2 ± 0.4 ^AB^	16.6 ± 0.2 ^AC^	18.9 ± 0.8 ^B^
**16:1n-9**	0.4 ± 0.0 ^ABD^	0.2 ± 0.0 ^C^	0.3 ± 0.0 ^CD^	0.4 ± 0.0 ^AC^	0.5 ± 0.1 ^B^	0.2 ± 0.0 ^C^	0.4 ± 0.1 ^ABD^
**16:1n-7**	6.6 ± 0.2 ^B^	17.4 ± 0.5 ^D^	10.1 ± 0.3 ^AC^	13.4 ± 1.0 ^ACE^	11.8 ± 0.8 ^C^	15.2 ± 0.4 ^DE^	9.7 ± 1.4 ^BC^
**15-17BFA**	1.8 ± 0.0 ^A^	1.0 ± 0.0 ^CD^	1.8 ± 0.1 ^A^	1.3 ± 0.0 ^AC^	2.5 ± 0.2 ^B^	0.6 ± 0.0 ^D^	1.0 ± 0.1 ^CD^
**16PUFA**	0.2 ± 0.0 ^B^	4.6 ± 0.2 ^C^	2.3 ± 0.2 ^DE^	1.5 ± 0.2 ^AD^	1.3 ± 0.1 ^A^	2.9 ± 0.1 ^E^	1.5 ± 0.4 ^AD^
**17:0**	0.2 ± 0.0 ^A^	0.3 ± 0.0 ^D^	0.4 ± 0.0 ^B^	0.2 ± 0.0 ^AD^	0.4 ± 0.0 ^B^	0.1 ± 0.0 ^C^	0.2 ± 0.0 ^A^
**18:0**	3.2 ± 0.1 ^C^	1.9 ± 0.0 ^D^	2.4 ± 0.1 ^A^	2.5 ± 0.2 ^AB^	3.0 ± 0.1 ^BC^	2.8 ± 0.0 ^AB^	2.5 ± 0.2 ^A^
**18:1n-9**	17.9 ± 0.1 ^AC^	19.8 ± 0.2 ^C^	16.1 ± 0.4 ^A^	16.8 ± 0.8 ^AC^	16.3 ± 0.7 ^A^	11.7 ± 0.6 ^B^	12.3 ± 1.0 ^B^
**18:1n-7**	3.1 ± 0.0 ^C^	3.9 ± 0.1 ^AC^	4.0 ± 0.3 ^AC^	4.3 ± 0.2 ^AB^	5.1 ± 0.1 ^B^	4.6 ± 0.1 ^AB^	3.7 ± 0.5 ^AC^
**18:2n-6**	3.0 ± 0.1 ^AC^	2.1 ± 0.1 ^A^	2.8 ± 0.1 ^AC^	2.7 ± 0.2 ^AC^	4.7 ± 0.6 ^B^	3.7 ± 0.1 ^BC^	2.7 ± 0.3 ^AC^
**18:3n-3**	2.6 ± 0.1 ^AC^	1.3 ± 0.0 ^A^	2.9 ± 0.2 ^BC^	2.2 ± 0.2 ^AC^	3.8 ± 0.6 ^B^	2.7 ± 0.2 ^AB^	1.6 ± 0.1 ^AC^
**18:4n-3**	1.7 ± 0.0	1.8 ± 0.1	1.8 ± 0.1	1.6 ± 0.1	1.5 ± 0.2	1.4 ± 0.2	1.3 ± 0.2
∑20:1 *	1.6 ± 0.0 ^AD^	1.2 ± 0.1 ^BCD^	2.3 ± 0.1 ^A^	2.1 ± 0.9 ^ABC^	1.7 ± 0.3 ^AB^	0.6 ± 0.0 ^C^	0.8 ± 0.1 ^BC^
**20:2n-6**	1.0 ± 0.0 ^C^	0.3 ± 0.0 ^A^	0.6 ± 0.0 ^B^	0.4 ± 0.0 ^A^	0.6 ± 0.0 ^B^	0.3 ± 0.0 ^A^	0.4 ± 0.1 ^A^
**20:4n-6**	1.9 ± 0.0 ^AC^	1.6 ± 0.0 ^CD^	2.4 ± 0.1 ^B^	2.3 ± 0.3 ^AB^	2.8 ± 0.1 ^B^	1.4 ± 0.1 ^D^	2.6 ± 0.2 ^B^
20:3n-3	1.5 ± 0.0 ^B^	0.2 ± 0.0 ^C^	0.6 ± 0.0 ^AB^	0.4 ± 0.0 ^ABC^	0.3 ± 0.0 ^AC^	0.2 ± 0.0 ^C^	0.4 ± 0.1 ^AC^
20:4n-3	2.9 ± 0.0 ^A^	0.7 ± 0.0 ^BC^	1.2 ± 0.1 ^AC^	1.1 ± 0.1 ^AC^	0.6 ± 0.0 ^B^	0.8 ± 0.0 ^BC^	0.8 ± 0.1 ^BC^
20:5n-3	4.8 ± 0.1 ^C^	10.4 ± 0.2 ^A^	9.6 ± 0.2 ^AB^	7.4 ± 0.7 ^ABC^	6.5 ± 0.3 ^BC^	10.2 ± 0.1 ^A^	10.3 ± 0.3 ^A^
**22:5n-6**	1.2 ± 0.0 ^B^	0.3 ± 0.0 ^C^	1.3 ± 0.1 ^B^	1.0 ± 0.1 ^AB^	0.8 ± 0.1 ^A^	0.3 ± 0.0 ^C^	0.8 ± 0.1 ^A^
22:4n-3	1.5 ± 0.1 ^C^	0.0 ± 0.0 ^AB^	0.1 ± 0.0 ^AC^	0.2 ± 0.1 ^AC^	0.0 ± 0.0 ^B^	0.0 ± 0.0 ^AB^	0.1 ± 0.0 ^ABC^
**22:5n-3**	3.0 ± 0.1 ^CD^	2.5 ± 0.1 ^AC^	2.5 ± 0.1 ^AC^	2.3 ± 0.1 ^A^	1.7 ± 0.1 ^B^	3.0 ± 0.0 ^D^	2.4 ± 0.1 ^A^
**22:6n-3**	12.1 ± 0.1 ^AC^	6.3 ± 0.2 ^D^	9.6 ± 0.6 ^ADE^	11.3 ± 1.1 ^ACD^	7.7 ± 0.9 ^AD^	14.4 ± 1.5 ^BCE^	20.1 ± 3.0 ^B^
24PUFA	4.3 ± 0.3 ^B^	1.0 ± 0.1 ^AB^	1.1 ± 0.1 ^BC^	1.0 ± 0.2 ^AC^	0.7 ± 0.0 ^A^	0.8 ± 0.0 ^A^	0.6 ± 0.1 ^AC^

* sum of 20:1n-11, 20:1n-9 and 20:1n-7, here and in other Tables.

**Table 4:** Mean levels of fatty acids (% of the total) and total content of fatty acids (Sum FA, mg g^−1^ wet weight) in polar lipids of Salmoniformes species: charr—*S. drjagini* from Sobachye Lake; whitefish—*C. lavaretus* from Sobachye Lake; muksun—*C. muksun* from Pyasino Lake; inconnu—*S. leucichthys nelma* from Pyasino Lake; broad whitefish—*C. nasus* from Pyasino Lake; round whitefish—*P. cylindraceum* from Sobachye Lake; whitefish nd—the non-identified form of *C. lavaretus* from Sobachye Lake. Cases (fatty acids) with normal distribution are given in bold. Means labeled with the same letter are not significantly different at *P* < 0.05 after ANOVA post hoc Tukey HSD test (cases with normal distribution) or Kruskal–Wallis test. If ANOVA is insignificant, letters are absent.

Fatty Acid	Charr	Whitefish	Muksun	Inconnu	Broad Whitefish	Round Whitefish	Whitefish nd
**14:0**	1.2 ± 0.1 ^AB^	0.9 ± 0.0 ^A^	1.1 ± 0.1 ^AB^	1.5 ± 0.2 ^B^	1.2 ± 0.1 ^AB^	0.9 ± 0.1 ^A^	1.4 ± 0.1 ^B^
**15:0**	0.2 ± 0.0 ^A^	0.2 ± 0.0 ^A^	0.4 ± 0.0 ^CD^	0.3 ± 0.0 ^BC^	0.5 ± 0.0 ^D^	0.2 ± 0.0 ^A^	0.3 ± 0.0 ^AB^
**16:0**	24.2 ± 0.4 ^A^	29.4 ± 0.5 ^BC^	26.3 ± 0.6 ^AB^	25.9 ± 0.5 ^AB^	27.3 ± 0.8 ^AB^	29.1 ± 1.3 ^BC^	31.7 ± 1.1 ^C^
**16:1n-9**	0.3 ± 0.0 ^AB^	0.1 ± 0.0 ^A^	0.3 ± 0.0 ^BC^	0.2 ± 0.1 ^AB^	0.5 ± 0.1 ^C^	0.2 ± 0.0 ^AB^	0.3 ± 0.1 ^BC^
**16:1n-7**	1.3 ± 0.1 ^A^	2.2 ± 0.1 ^BC^	1.9 ± 0.2 ^AB^	2.3 ± 0.1 ^B^	2.9 ± 0.2 ^B^	4.1 ± 0.2 ^D^	3.0 ± 0.2 ^C^
**15-17BFA**	0.6 ± 0.0 ^C^	0.1 ± 0.0 ^A^	0.4 ± 0.0 ^BC^	0.3 ± 0.0 ^AB^	0.6 ± 0.1 ^C^	0.2 ± 0.0 ^AB^	0.3 ± 0.1 ^AB^
**16PUFA**	0.0 ± 0.0 ^A^	0.2 ± 0.0 ^B^	0.1 ± 0.1 ^AB^	0.1 ± 0.0 ^AB^	0.1 ± 0.0 ^AB^	0.3 ± 0.0 ^C^	0.1 ± 0.0 ^AB^
**17:0**	0.2 ± 0.0 ^A^	0.2 ± 0.0 ^AB^	0.3 ± 0.0 ^B^	0.2 ± 0.0 ^AB^	0.3 ± 0.0 ^B^	0.1 ± 0.0 ^A^	0.2 ± 0.0 ^A^
**18:0**	2.8 ± 0.1 ^AB^	2.1 ± 0.1 ^A^	3.0 ± 0.1 ^B^	2.9 ± 0.3 ^AB^	2.2 ± 0.2 ^A^	3.4 ± 0.2 ^B^	2.8 ± 0.3 ^AB^
**18:1n-9**	6.2 ± 0.3 ^AB^	6.5 ± 0.2 ^AB^	6.6 ± 0.4 ^AB^	7.8 ± 0.4 ^B^	6.1 ± 0.5 ^A^	5.6 ± 0.3 ^A^	6.9 ± 0.3 ^AB^
**18:1n-7**	1.6 ± 0.1 ^A^	1.7 ± 0.1 ^AB^	2.2 ± 0.2 ^BC^	2.4 ± 0.1 ^C^	2.5 ± 0.1 ^C^	3.3 ± 0.2 ^D^	2.4 ± 0.3 ^C^
**18:2n-6**	0.7 ± 0.0 ^A^	0.8 ± 0.0 ^A^	1.0 ± 0.0 ^A^	1.1 ± 0.1 ^A^	2.5 ± 0.4 ^B^	1.6 ± 0.0 ^A^	1.4 ± 0.3 ^A^
18:3n-3	0.6 ± 0.0 ^AB^	0.4 ± 0.0 ^A^	1.0 ± 0.1 ^ABC^	1.0 ± 0.2 ^ABC^	2.1 ± 0.4 ^C^	1.3 ± 0.1 ^BC^	0.7 ± 0.1 ^ABC^
**18:4n-3**	0.1 ± 0.0 ^AB^	0.0 ± 0.0 ^A^	0.2 ± 0.0 ^B^	0.2 ± 0.0 ^AB^	0.1 ± 0.0 ^AB^	0.3 ± 0.0 ^B^	0.2 ± 0.1 ^AB^
**∑20:1**	0.2 ± 0.0 ^BC^	0.1 ± 0.0 ^A^	0.3 ± 0.0 ^C^	0.1 ± 0.0 ^ABC^	0.1 ± 0.0 ^AB^	0.2 ± 0.0 ^ABC^	0.2 ± 0.1 ^AC^
**20:2n-6**	0.2 ± 0.0 ^BC^	0.0 ± 0.0 ^A^	0.1 ± 0.0 ^AB^	0.1 ± 0.0 ^A^	0.2 ± 0.0 ^C^	0.1 ± 0.0 ^AC^	0.1 ± 0.0 ^AB^
**20:4n-6**	3.9 ± 0.1 ^BC^	3.0 ± 0.1 ^AB^	4.1 ± 0.2 ^C^	3.8 ± 0.3 ^BC^	5.6 ± 0.3 ^D^	2.4 ± 0.1 ^A^	3.2 ± 0.2 ^ABC^
**20:3n-3**	0.5 ± 0.0 ^C^	0.1 ± 0.0 ^A^	0.2 ± 0.0 ^B^	0.1 ± 0.0 ^AB^	0.2 ± 0.0 ^B^	0.1 ± 0.0 ^AB^	0.1 ± 0.0 ^AB^
20:4n-3	1.1 ± 0.0 ^B^	0.3 ± 0.0 ^A^	0.5 ± 0.0 ^AB^	0.5 ± 0.1 ^AB^	0.4 ± 0.1 ^A^	0.5 ± 0.0 ^AB^	0.4 ± 0.0 ^A^
**20:5n-3**	7.9 ± 0.2 ^A^	16.6 ± 0.6 ^D^	12.5 ± 0.3 ^BC^	11.2 ± 1.1 ^BC^	10.9 ± 0.4 ^B^	13.6 ± 1.0 ^C^	10.0 ± 0.5 ^AB^
**22:5n-6**	2.4 ± 0.1 ^C^	0.6 ± 0.0 ^A^	2.0 ± 0.3 ^C^	1.7 ± 0.2 ^BC^	2.2 ± 0.2 ^C^	0.4 ± 0.0 ^A^	0.9 ± 0.1 ^AB^
22:4n-3	0.2 ± 0.0 ^B^	0.0 ± 0.0 ^A^	0.0 ± 0.0 ^A^	0.0 ± 0.0 ^A^	0.0 ± 0.0 ^A^	0.0 ± 0.0 ^A^	0.0 ± 0.0 ^A^
**22:5n-3**	2.5 ± 0.0 ^A^	2.7 ± 0.2 ^AB^	2.5 ± 0.1 ^A^	2.4 ± 0.3 ^A^	2.6 ± 0.1 ^AB^	3.2 ± 0.3 ^B^	2.1 ± 0.1 ^A^
**22:6n-3**	39.9 ± 0.5 ^D^	31.1 ± 0.7 ^BC^	31.8 ± 0.5 ^C^	32.4 ± 0.9 ^C^	26.4 ± 0.6 ^A^	27.9 ± 0.7 ^AB^	30.1 ± 1.1 ^BC^
24PUFA	0.3 ± 0.1	0.0 ± 0.0	0.0 ± 0.0	0.4 ± 0.4	0.1 ± 0.0	0.0 ± 0.0	0.0 ± 0.0
*Sum FA*	*3.0 ± 0.2 ^AB^*	*3.3 ± 0.5 ^AB^*	*3.2 ± 0.9 ^AB^*	*2.6 ± 0.2 ^AB^*	*3.4 ± 0.2 ^B^*	*3.2 ± 0.7 ^AB^*	*2.1 ± 0.2 ^A^*

**Table 5:** Mean levels of fatty acids (% of the total) and total content of fatty acids (Sum FA, mg g^−1^ wet weight) in triacylglycerols of Salmoniformes species: charr—*S. drjagini* from Sobachye Lake; whitefish—*C. lavaretus* from Sobachye Lake; muksun—*C. muksun* from Pyasino Lake; inconnu—*S. leucichthys nelma* from Pyasino Lake; broad whitefish—*C. nasus* from Pyasino Lake; round whitefish—*P. cylindraceum* from Sobachye Lake; whitefish nd—the non-identified form of *C. lavaretus* from Sobachye Lake. Cases (fatty acids) with normal distribution are given in bold. Means labeled with the same letter are not significantly different at *P* < 0.05 after ANOVA post hoc Tukey HSD test (cases with normal distribution) or Kruskal–Wallis test. If ANOVA is insignificant, letters are absent.

Fatty Acid	Charr	Whitefish	Muksun	Inconnu	Broad Whitefish	Round Whitefish	Whitefish nd
**14:0**	4.4 ± 0.1 ^CD^	3.3 ± 0.1 ^B^	4.8 ± 0.3 ^D^	3.9 ± 0.0 ^BC^	2.7 ± 0.2 ^A^	3.1 ± 0.2 ^B^	3.5 ± 0.4 ^ABC^
**15:0**	0.3 ± 0.0 ^B^	0.2 ± 0.0 ^AB^	0.5 ± 0.0 ^C^	0.4 ± 0.0 ^BC^	0.5 ± 0.1 ^C^	0.2 ± 0.0 ^A^	0.3 ± 0.0 ^AB^
**16:0**	16.7 ± 0.4 ^C^	14.6 ± 0.2 ^AB^	14.6 ± 0.8 ^AB^	15.9 ± 0.7 ^B^	17.4 ± 0.3 ^C^	12.9 ± 0.4 ^A^	15.7 ± 0.5 ^B^
16:1n-9	0.8 ± 0.3 ^B^	0.3 ± 0.0 ^A^	0.4 ± 0.0 ^AB^	0.4 ± 0.1 ^AB^	0.8 ± 0.1 ^B^	0.3 ± 0.0 ^A^	0.5 ± 0.1 ^AB^
**16:1n-7**	7.9 ± 0.4 ^A^	19.4 ± 0.8 ^C^	12.6 ± 1.3 ^B^	15.9 ± 1.4 ^BC^	13.2 ± 0.7 ^B^	24.5 ± 1.2 ^D^	15.7 ± 0.9 ^BC^
15-17BFA	2.0 ± 0.2 ^BCD^	1.0 ± 0.0 ^AB^	1.9 ± 0.1 ^CD^	1.1 ± 0.1 ^ABC^	2.8 ± 0.4 ^D^	0.7 ± 0.1 ^A^	1.4 ± 0.1 ^AB^
**16PUFA**	0.4 ± 0.1 ^A^	4.8 ± 0.2 ^C^	3.0 ± 0.6 ^B^	1.6 ± 0.4 ^AB^	1.3 ± 0.2 ^AB^	5.4 ± 0.7 ^C^	2.2 ± 0.5 ^B^
**17:0**	0.2 ± 0.0 ^AB^	0.3 ± 0.0 ^BC^	0.3 ± 0.0 ^C^	0.2 ± 0.0 ^AB^	0.5 ± 0.0 ^D^	0.1 ± 0.0 ^A^	0.3 ± 0.0 ^BC^
**18:0**	3.4 ± 0.1 ^C^	1.9 ± 0.0 ^A^	2.4 ± 0.1 ^A^	2.5 ± 0.3 ^AB^	3.2 ± 0.1 ^BC^	2.5 ± 0.1 ^A^	2.6 ± 0.3 ^A^
**18:1n-9**	19.2 ± 0.6 ^BC^	21.5 ± 0.4 ^C^	16.8 ± 1.0 ^AB^	18.9 ± 1.4 ^B^	17.8 ± 0.8 ^AB^	14.6 ± 0.8 ^A^	18.3 ± 0.6 ^B^
**18:1n-7**	2.9 ± 0.4 ^A^	4.2 ± 0.1 ^AB^	4.6 ± 0.4 ^BC^	4.9 ± 0.2 ^BC^	5.9 ± 0.3 ^C^	5.9 ± 0.4 ^C^	4.8 ± 0.5 ^BC^
**18:2n-6**	3.2 ± 0.1 ^AB^	2.2 ± 0.1 ^A^	3.0 ± 0.1 ^AB^	3.0 ± 0.4 ^AB^	5.4 ± 0.6 ^D^	5.1 ± 0.1 ^CD^	3.9 ± 0.3 ^BC^
**18:3n-3**	2.6 ± 0.1 ^AB^	1.4 ± 0.1 ^A^	3.0 ± 0.2 ^BC^	2.4 ± 0.3 ^A^	4.0 ± 0.5 ^C^	3.6 ± 0.4 ^BC^	2.2 ± 0.2 ^AB^
**18:4n-3**	1.6 ± 0.1	1.8 ± 0.1	1.8 ± 0.1	1.7 ± 0.2	1.5 ± 0.2	1.9 ± 0.3	2.1 ± 0.4
∑20:1	1.3 ± 0.0 ^BC^	1.0 ± 0.1 ^AB^	1.9 ± 0.2 ^C^	2.0 ± 0.9 ^ABC^	1.7 ± 0.3 ^BC^	0.7 ± 0.1 ^A^	1.3 ± 0.1 ^ABC^
**20:2n-6**	0.9 ± 0.0 ^C^	0.2 ± 0.0 ^A^	0.6 ± 0.0 ^B^	0.4 ± 0.0 ^AB^	0.4 ± 0.1 ^AB^	0.3 ± 0.0 ^AB^	0.5 ± 0.1 ^B^
**20:4n-6**	1.7 ± 0.0 ^BC^	1.4 ± 0.0 ^B^	2.1 ± 0.1 ^D^	1.9 ± 0.2 ^CD^	2.3 ± 0.1 ^D^	0.6 ± 0.0 ^A^	1.7 ± 0.1 ^BCD^
20:3n-3	1.4 ± 0.0 ^C^	0.1 ± 0.0 ^A^	0.5 ± 0.1 ^BC^	0.4 ± 0.0 ^ABC^	0.3 ± 0.0 ^AB^	0.2 ± 0.0 ^AB^	0.4 ± 0.1 ^AB^
20:4n-3	2.7 ± 0.1 ^C^	0.7 ± 0.1 ^AB^	1.2 ± 0.1 ^BC^	1.2 ± 0.1 ^ABC^	0.6 ± 0.0 ^A^	0.9 ± 0.1 ^AB^	0.9 ± 0.1 ^AB^
**20:5n-3**	4.4 ± 0.1 ^A^	9.4 ± 0.2 ^D^	9.0 ± 0.3 ^D^	6.5 ± 1.0 ^BC^	5.3 ± 0.4 ^AB^	7.0 ± 0.5 ^BC^	8.6 ± 0.5 ^CD^
**22:5n-6**	0.9 ± 0.0 ^D^	0.2 ± 0.0 ^AB^	1.0 ± 0.1 ^D^	0.7 ± 0.1 ^CD^	0.5 ± 0.1 ^BC^	0.0 ± 0.0 ^A^	0.4 ± 0.1 ^AB^
22:4n-3	1.3 ± 0.0 ^B^	0.0 ± 0.0 ^A^	0.1 ± 0.0 ^A^	0.2 ± 0.1 ^A^	0.0 ± 0.0 ^A^	0.0 ± 0.0 ^A^	0.1 ± 0.0 ^A^
**22:5n-3**	2.7 ± 0.1 ^B^	2.2 ± 0.1 ^B^	2.4 ± 0.2 ^B^	2.1 ± 0.2 ^B^	1.4 ± 0.1 ^A^	2.3 ± 0.1 ^B^	2.1 ± 0.2 ^B^
**22:6n-3**	9.9 ± 0.2 ^C^	4.1 ± 0.2 ^B^	6.4 ± 0.3 ^C^	7.8 ± 0.8 ^C^	4.1 ± 0.3 ^B^	2.4 ± 0.2 ^A^	6.6 ± 0.5 ^C^
24PUFA	2.9 ± 0.1 ^B^	0.7 ± 0.1 ^A^	0.9 ± 0.1 ^AB^	0.6 ± 0.3 ^A^	0.7 ± 0.0 ^A^	0.6 ± 0.1 ^A^	0.7 ± 0.1 ^A^
*Sum FA*	*30.3 ± 4.7 ^AB^*	*33.2 ± 6.9 ^AB^*	*41.6 ± 15.0 ^B^*	*18.7 ± 7.0 ^AB^*	*19.5 ± 7.0 ^AB^*	*4.9 ± 1.2 ^A^*	*2.0 ± 0.5 ^A^*
